# C-reactive Protein Variation and Its Usefulness in the Prognostication and Monitoring of Patients With Pneumococcal Pneumonia

**DOI:** 10.7759/cureus.72112

**Published:** 2024-10-22

**Authors:** André Gomes, Rui Ribeiro, Filipe Froes, Paulo Mergulhão, João Gonçalves Pereira

**Affiliations:** 1 Intensive Care Unit, Hospital da CUF, Porto, PRT; 2 Medicine, Grupo de Infecção e Sépsis, Porto, PRT; 3 Internal Medicine, Centro Hospitalar Universitário de São João - EPE, Porto, PRT; 4 Intensive Care Unit, Hospital Pulido Valente - Centro Hospitalar Universitário Lisboa Norte, Lisbon, PRT; 5 Medicine, Grupo de Infecção e Sepsis, Porto, PRT; 6 Intensive Care Unit, Hospital Lusíadas Porto, Porto, PRT; 7 Intensive Care Unit, Hospital Vila Franca de Xira, Vila Franca de Xira, PRT

**Keywords:** age-related mortality, c-reactive protein, pneumococcus, pneumonia, prognostic

## Abstract

Background and objective

Community-acquired pneumonia (CAP) is a prevalent and life-threatening infection that causes significant morbidity and mortality. Biomarkers, such as C-reactive protein (CRP), can help to diagnose, monitor, and prognose patients with this condition. This study aimed to analyze the disease course, the CRP peak concentration, its relationship with prognosis, and its variation in hospitalized patients with pneumococcal CAP.

Methodology

This study included 797 patients diagnosed with pneumococcal CAP and admitted over four years to four different Portuguese hospitals, either to the ICU or the general ward.

Results

Although CRP peak concentration was not a good predictor of overall hospital mortality, higher peak concentration in older patients (>60 years) was associated with a dismal hospital prognosis. In contrast, younger patients who survived hospital discharge had a non-significant higher peak CRP concentration. A faster time until CRP decreased to at least half of its peak value also correlated with favorable outcomes after adjusting for age and bacteremia [failure to achieve a 50% decrease was associated with an adjusted hazard ratio (HR) for hospital mortality of 6.45; 95% confidence interval (CI): 4.30-9.69].

Conclusions

Based on our findings, CRP may be a useful biomarker in the hospital setting for diagnosing and monitoring patients with pneumococcal CAP. Clinicians must be aware of its unique properties, clinical applications, and varying behaviors according to patient age groups.

## Introduction

Community-acquired pneumonia (CAP) is a prevalent and potentially life-threatening respiratory infection that affects individuals of all ages worldwide. Streptococcus pneumoniae, or pneumococcus, is the most common agent associated with this condition [1-3]. Pneumococcus can invade the lower respiratory tract and evade the host immune response, making it a key player in CAP pathogenesis [4,5]. Older patients are known to be at increased risk of complications, probably due to comorbidities, frailty, and immunosenescence [6]. Diagnosing pneumococcal CAP and assessing its severity is challenging due to its wide range of clinical symptoms and varying individual responses. While traditional diagnostic tools, such as blood and sputum cultures, are still used, they provide limited insights into disease prognosis and patient monitoring. Hence, there is a growing interest in employing biomarkers, such as C-reactive protein (CRP), to address these gaps [7-12].

CRP is an acute-phase protein that the liver synthesizes in response to inflammation, particularly infection. Monitoring CRP kinetics can provide valuable information about disease progression and treatment effectiveness [13]. In light of this, we conducted a large, epidemiological, retrospective, multicentre study involving patients admitted to various hospitals with pneumococcal CAP. We aimed to assess the disease evolution, the CRP peak concentration, its relationship with prognosis, and its variation.

## Materials and methods

This retrospective, multicentre cohort study included all consecutive patients admitted with pneumococcal CAP to any of the four participating centers, between 2015 and 2018. The study protocol and the general characteristics of the population have been published elsewhere [14]. In brief, patients with either a positive urinary pneumococcal antigen or a positive blood culture for Streptococcus pneumoniae were segregated for further analysis. One of the local investigators reviewed the patients' clinical, radiological, and laboratory features, to determine if the same was compatible with CAP due to Streptococcus pneumoniae. Patients deemed not to be infected or to have another focus of infection were excluded from further analysis. Besides, patients younger than 18 years at admission to the hospital were also excluded.

Demographic data (gender, age on hospital admission, hospital length of stay, ICU admission, mortality, hospital readmission, or death during the first year of follow-up); clinical data (comorbidities, previous functional status, sepsis, pleural effusion, multilobe involvement); laboratory results (including all available CRP measurements); therapeutics (invasive or non-invasive mechanical ventilation, renal replacement therapy, antibiotics, steroids) were collected in a dedicated database. The study was approved in all participating centers by the respective ethical boards. Informed consent was waived due to the study's retrospective, observational-only nature.

Evaluation of all CRP measurements was performed. Peak CRP concentration was identified for all patients. The time lag (in days) between the individual CRP peak concentration and a CRP concentration below at least 50% of that same peak was computed. Patients were divided into three groups according to this time frame: <4 days, ≥4 days, and those who did not achieve that target. Mean CRP peak concentration was categorized according to 10-year age groups and survivorship and plotted into a graph. After visual inspection, the population was split into two groups (up to 60 years and older than 60 years). Differences in CRP peak concentration between hospital survivors and non-survivors were computed for the whole population and age groups.

Statistical analysis

The features of all included patients were evaluated through descriptive analysis. According to data distribution, continuous variables were reported as mean ± standard deviation (SD) or median [interquartile range (IQR)]. Categorical variables were reported as counts (percentage). Baseline demographics and clinical characteristics were compared between survivors and non-survivors, using the student's t-test or Mann-Whitney U test for continuous variables, according to data distribution, and Chi-square for categorical variables. Analysis of variance was performed with the ANOVA test. Odds ratio (OR) with 95% confidence intervals (CIs) were computed. According to the kinetic of CRP reduction, survival between the three defined groups was calculated using the Cox proportional hazards ratio (HR), with 95% CI, adjusted for bacteremia and age (which proved to be the most important single variables associated with mortality). Statistical analysis was performed using IBM SPSS Statistics v.28.0 (IBM, Armonk, NY). All statistics were two-tailed, and the significance level was set at p<0.05.

## Results

The initial study population comprised 797 patients; the cohort was mainly male (n=427, 53.6%) and had a mean age of 72.3 ±16.5 years. More than 90% (n=739) had at least one significant comorbidity: arterial hypertension (n=378, 47.4%), diabetes mellitus (n=216, 27.1%), and congestive heart failure (n=210, 26.3%) (Table 1). All patients had undergone at least one CRP test and two or more measurements were available for 752.

**Table 1:** Demographic and clinical characteristics of the study population (n=797) CKD: chronic kidney disease; COPD: chronic obstructive pulmonary disease; HIV: human immunodeficiency vírus; IMV: invasive mechanical ventilation; NIV: non-invasive ventilation; SD: standard deviation

Variables	Values
Age, years, mean ±SD	72.3 ±16.4
Male, n (%)	427 (53,6)
Functional status	
Bedridden, n (%)	139 (17.4)
Dependent, n (%)	173 (21.7)
Autonomous, n (%)	485 (60.8)
Comorbidities	
Diabetes, n (%)	216 (27.1)
Hypertension, n (%)	378 (47.4)
Cerebrovascular disease, n (%)	96 (12.0)
Atrial fibrillation, n (%)	158 (19.8)
Ischemic heart disease, n (%)	74 (9.3)
Heart failure, n (%)	210 (26.3)
COPD, n (%)	137 (17.2)
CKD, n (%)	111 (13.9)
Liver disease, n (%)	41 (5.1)
HIV, n (%)	27 (3.4)
Alcohol abuse, n (%)	77 (9.6)
ICU admission, n (%)	150 (18.8)
IMV, n (%)	53 (6.6)
NIV, n (%)	96 (12.0)
Bacteremia, n (%)	252 (31.6)
Pleural effusion	164 (20.6)
Multilobar involvement	300 (37.6)
Hospital length of stay, median (p25-p75)	9 (6-14)
Hospital mortality, n (%)	142 (17.8)
One-year mortality, n (%)	261 (32.7)

Bacteremia was present in approximately a third of the patients. Blood cultures of 465 (58.3%) patients were not collected on admission to the hospital, and this group had the lowest mortality (12.5%). Nearly a fifth of our population was admitted to the ICU. The hospital mortality was 16.2% (n=122) (Table 2). Patients discharged alive from the hospital were significantly younger and had a lower prevalence of bacteremia. No mean peak CRP concentration difference was found between survivors and non-survivors in the overall population (p=0.06) (Table 2).

**Table 2:** Hospital mortality and all-cause one-year mortality *Student's t-test; **Mann-Whitney U test; ^§^Chi-square test CRP: C-reactive protein; ICU: intensive care unit; IMV: invasive mechanical ventilation; LOS: length of stay; NIV: non-invasive ventilation; SD: standard deviation

	In-hospital mortality			One-year all-cause mortality		
	Survived (n=630)	Deceased (n=122)	P-value	Survived (n=517)	Deceased (n=235)	P-value
Age, years, mean (SD)	71.1 (16.7)	78.5 (13.6)	<0.001*	68.9 (17.1)	79.9 (11.9)	<0.001*
Male, n (%)	335 (53.2)	68 (55.7)	0.270^§^	274 (53)	124 (54.4)	0.629^§^
CRP peak concentration, mean (SD)	23.7 (12.3)	25.9 (13.0)	0.064*	24.2 (12.5)	23.7 (12.4)	0.592*
LOS, median (p25-p75)	9 (7-14)	8 (4-15)	<0.001**	9 (7-13)	10 (6-16)	0.498**
Bacteremia, n (%)	182 (28.9)	57 (46.7)	<0.001^§^	152 (29.4)	87 (37.0)	0.044^§^
ICU admission, n (%)	114 (18.1)	29 (23.8)	0.144^§^	105 (24.3)	38 (16.2)	0.180^§^
IMV, n (%)	37 (5.9)	11 (9.0)	0.195^§^	33 (6.4)	15 (6.4)	0.995^§^
NIV, n (%)	66 (10.5)	28 (23.0)	<0.001^§^	55 (10.6)	34 (66.6)	0.023^§^

**Figure 1:**
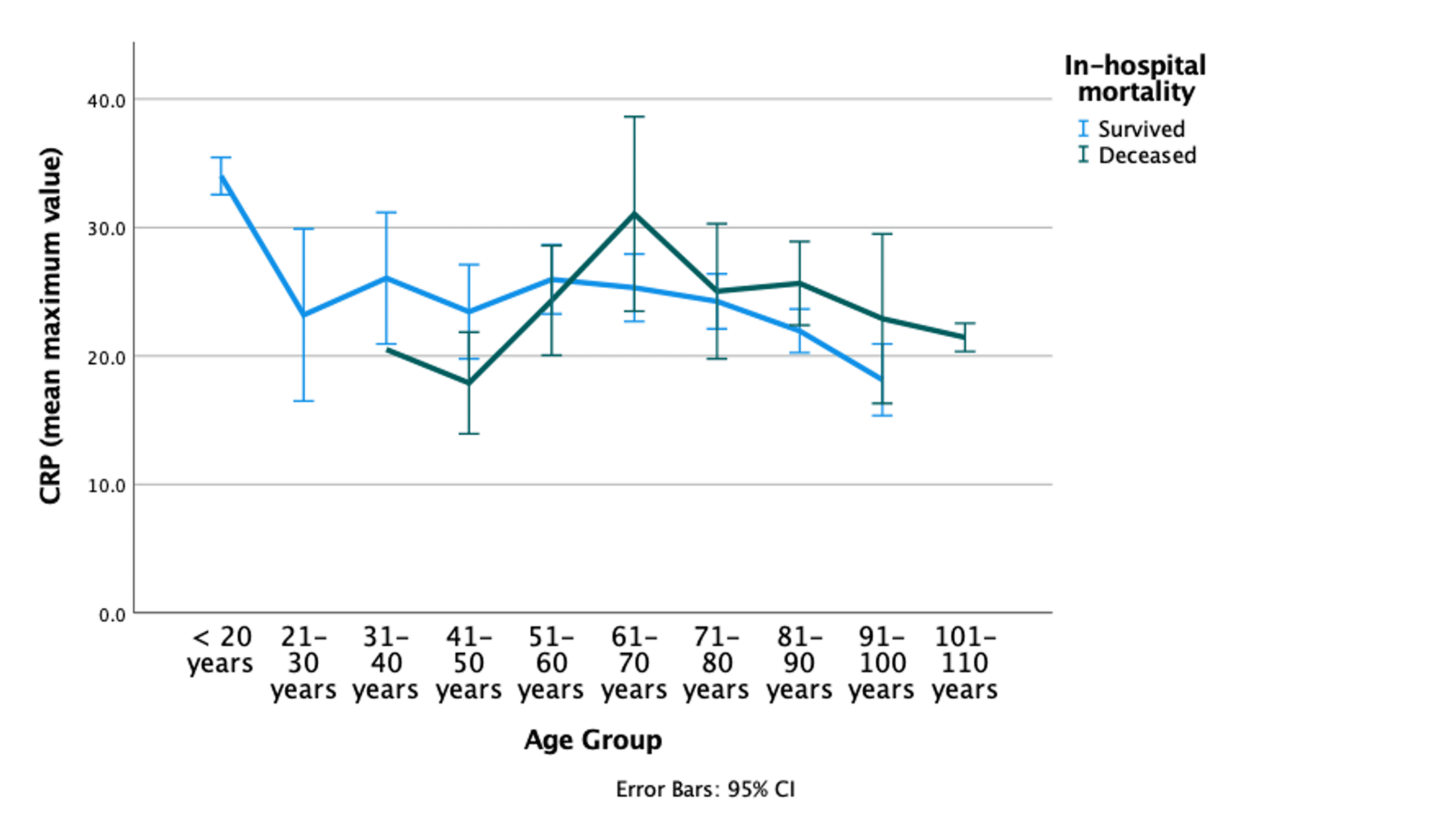
Comparison of CRP maximum values between survivors and non-survivors among all patients by age group In patients older than 60 years, the CRP mean peak concentration was higher in non-survivors (n=108) than survivors (n=467): 26.4 (SD: 13.5) vs. 23.0 (SD: 12.3), p=0.017 (student's t-test). However, this difference was not noted in younger patients: non-survivors [n=14; CRP mean peak concentration: 22.4 (SD: 7.2) vs. survivors (n=63) 25.7 (SD: 12.2), p=0.151 (student's t-test)] CI: confidence interval; CRP: C-reactive protein; SD: standard deviation

An association between age, peak CRP concentration, and survival was unveiled. In younger patients (up to 60 years of age), survivors had roughly 14% higher CRP peak concentration (Figure 1), although this difference was non-significant (p=0.15). In contrast, in older patients (≥60 years), non-survivors had a mean peak CRP concentration nearly 14% higher (p=0.017) (Figure 1).

**Figure 2:**
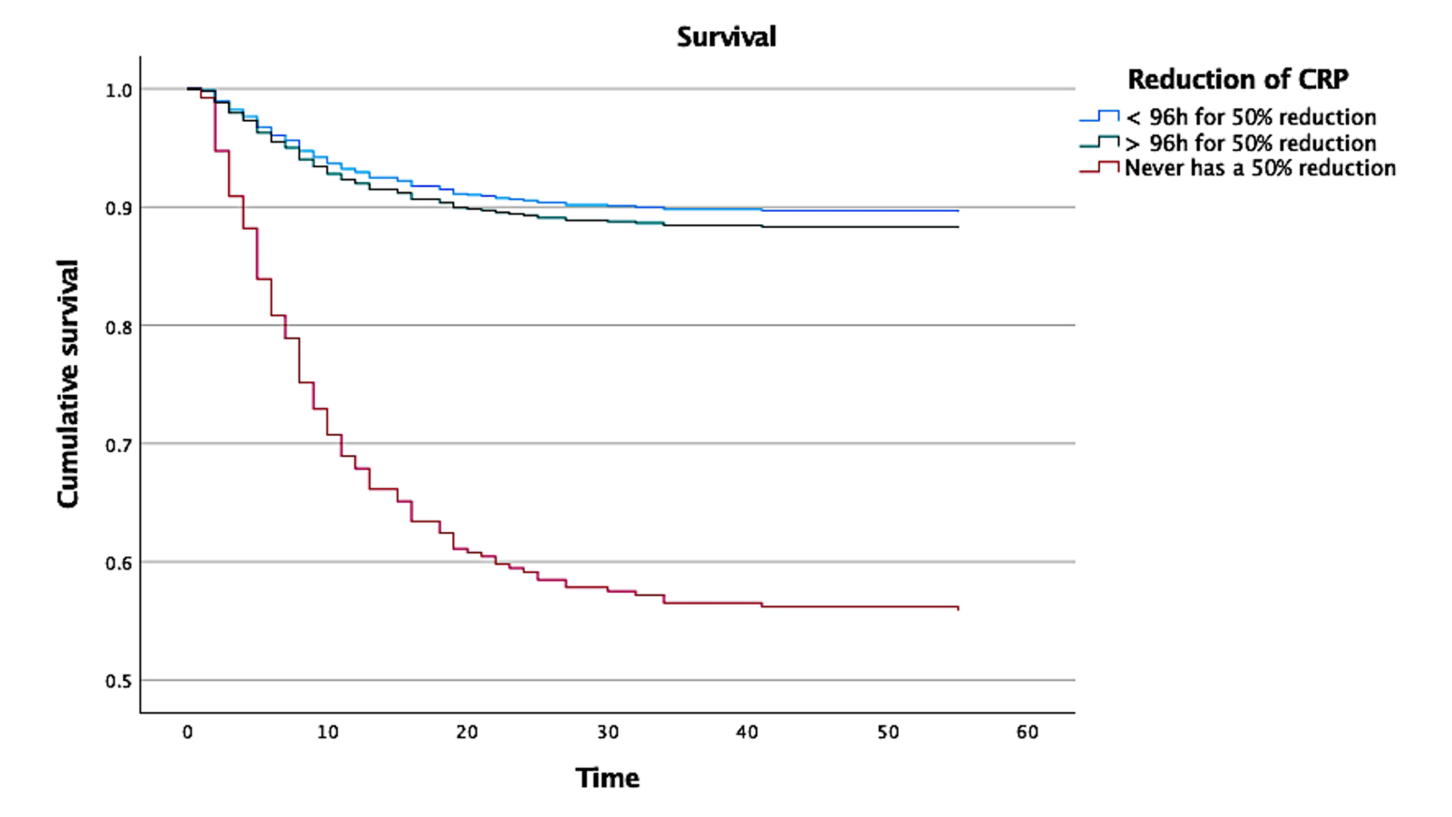
Survival curve for CRP reduction in less than 96 hours in comparison with that in >96 hours and that never reduced by 50% The HR (adjusted for age and the presence of bacteremia) for mortality in patients who achieved 50% CRP decrease more than 96 hours after the individual peak concentration was non-significant: 1.06 (95% CI: 0.64-1.75). However, for patients who never achieved this reduction, the adjusted HR was 6.45 (95% CI: 4.30-9.69). Reference: patients with a 50% CRP reduction before 96 hours CI: confidence interval; CRP: C-reactive protein; HR: hazard ratio

The time taken for a 50% reduction in individual peak CRP was less than four days in 472 (62.8%) of the studied patients, while 106 patients (14.1%) never achieved that reduction during their hospital stay. Patients with a faster decrease in CRP concentration had significantly lower hospital mortality (Figure 2) when compared with patients who achieved the same reduction after a longer period, or those who never completed it, the latter with HR (adjusted for age and the presence of bacteremia) of 6.45 (95% CI: 4.30-9.69).

**Figure 3:**
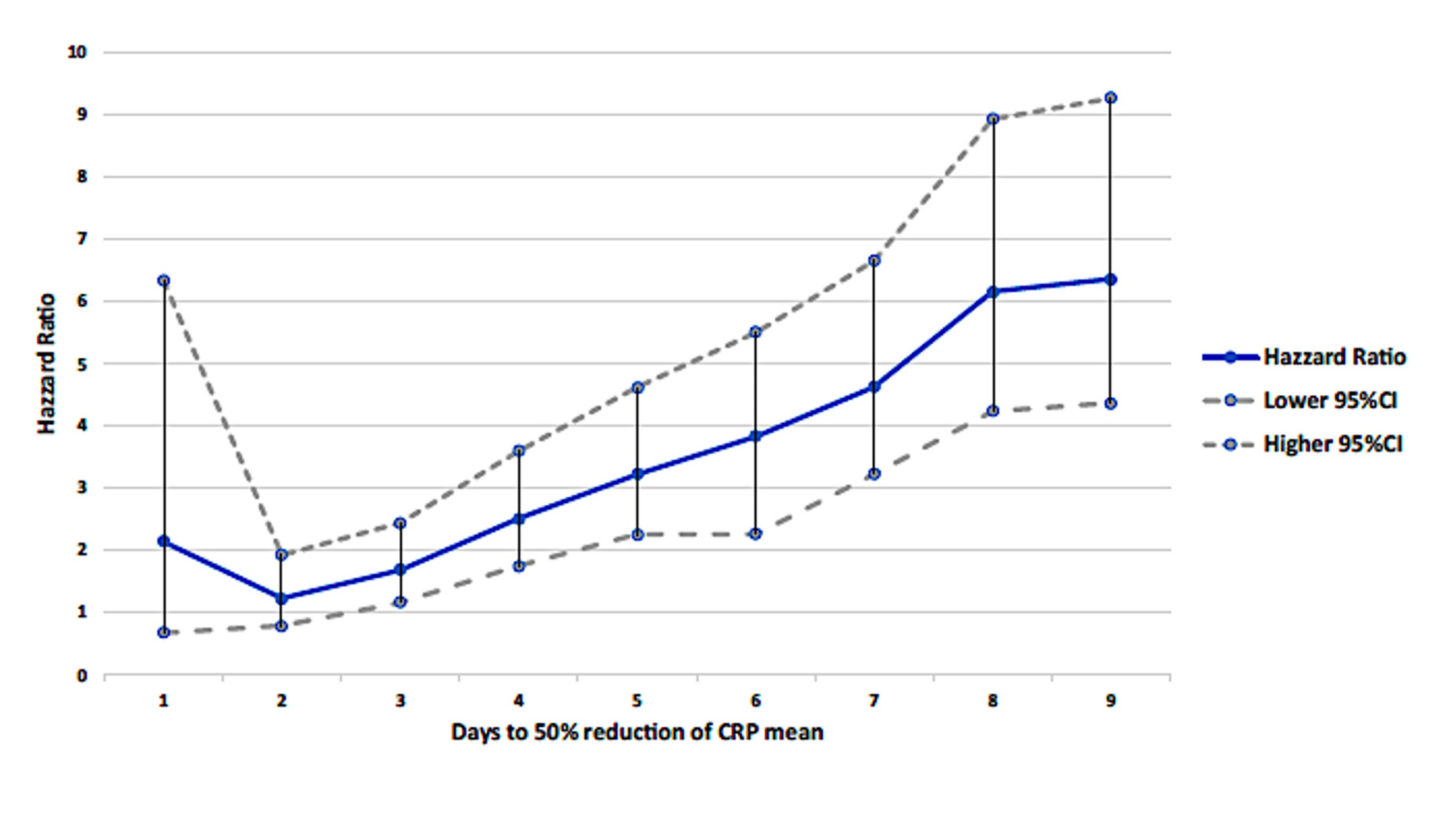
HR for hospital mortality calculated each day by comparing patients who achieved a 50% reduction in maximum CRP with those who did not (adjusted for age and presence of bacteremia) HR (adjusted for age and the presence of bacteremia) with a 95% CI of mortality for patients who achieved a 50% CRP decrease each day compared to those who did not achieve that reduction on the same day. A steady increase was noted from day two onwards, with no clear cut-off day. Vertical bars: 95% CI CI: confidence interval; CRP: C-reactive protein; HR: hazard ratio

As shown in Figure 3, we plotted the daily adjusted HR (with 95% CI) for hospital mortality, comparing, for each day, the patients who had already achieved a 50% reduction of their peak CRP with those who did not. A steady increase in adjusted HR for hospital mortality was noted from day two onwards.

## Discussion

In this multicentre epidemiological study, we evaluated the CRP dynamics in 752 patients admitted to the hospital with pneumococcal CAP. Our population was relatively old (mean age: 72.3, ±16.5 years), with a high prevalence of cardiovascular risk factors and disease. We found a biphasic relationship between the mean CRP peak concentration and hospital mortality. Accordingly, the high mean CRP peak concentration was associated with hospital mortality in patients older than 60 years, while in younger patients, we noted a trend toward an inverse relationship. Moreover, CRP concentration kinetics was also linked to prognosis: a decrease of more than 50% from the peak value before four days was associated with an increased survival rate. Interestingly, this benefit progressively decreased with the delay until this 50% decrease was achieved from the second day onwards, with no clear cut-off point.

Accordingly, CRP was not only helpful for CAP diagnosis but also for assessing clinical severity and aiding in outcome prediction, as previously proposed [3,13,15-18]. The relationship between CRP peak concentration and prognosis for a whole population has been a matter of controversy [17,19,20], and poor predictive values have been reported. Furthermore, as infected patients often have a poor prognosis, CRP may only be a surrogate for infection rather than a good prognosis marker [21]. In this study, we had a more homogeneous population, all with pneumococcal CAP. Again, the whole population's mean CRP peak concentration was not predictive of survival. However, we found a biphasic pattern: younger patients (<60 years old) who survived had higher CRP peak concentration, while the opposite trend was noted in older patients, as shown in Figure 1. This could be attributed to the pleiotropic inflammatory effects of CRP, i.e., defense against infection but also a pro-atherosclerotic impact: younger patients, with a lower cardiovascular risk, may benefit from the former, while the latter may lead to vascular complications in older patients [22]. Besides, in older age groups, the higher CRP may indicate a more severe general disease [23] or even other inflammatory conditions such as auto-inflammatory conditions, cardiovascular disease, or active neoplasia [18,24].

Several studies have shown a close link between CRP concentration kinetics (the speed at which this decrease occurs) and an improved prognosis, mainly in the ICU setting [25,26]. Failure to reduce CRP concentration to less than 50% after 72-96 hours has been associated with inappropriate antibiotic therapy and possible parapneumonic complications, such as empyema or bacteremia [8,13,27]. In our cohort, failure, or even delay, to decrease CRP concentration was associated with a steady increase in adjusted HR for hospital mortality from the second day onward, which is consistent with other studies, in the ICU setting [28-30], although we did not find any clear cut-off point.

Our study has some limitations. It was a retrospective study based only on clinical records. There was no systematic patient assessment on hospital admission, and many patients, especially with less severe presentations, may not have been included. Secondly, no protocolized assessment was made on patient admission in any of the included hospitals. Consequently, more than 50% of patients did not collect blood cultures. Thirdly, we included patients admitted over four years: although no substantial changes to severe CAP treatment have occurred during this period, some unknown biases may have been missed. Fourth, we addressed a relatively older population. Fifth, CRP concentration was not measured daily in all patients. Per our inclusion criteria, patients with a positive urinary pneumococcal antigen were selected if the clinical presentation aligned with CAP. However, due to the persistence of this antigen, patients admitted with CAP shortly after a pneumococcal infection may have been misclassified. Nevertheless, as our study mainly focused on the CRP concentration, we believe this to have little impact on our results.

Our study also has some significant strengths as well; it includes a large cohort of patients, all of them hospitalized adult individuals diagnosed with pneumococcal CAP, admitted to the ICU and the general ward. Their clinical severity was high, as indicated by the rate of ventilated patients, the mortality rate, and the significant number of patients with bacteremia, which facilitated the assessment of prognosis. Finally, we had a relatively homogeneous group.

## Conclusions

Based on our findings, in patients admitted to the hospital with documented pneumococcal CAP, the daily monitoring of CRP concentration may help identify the prognosis and evaluate clinical response to therapy. In patients older than 60 years, a higher CRP peak concentration was associated with a worse prognosis. On the contrary, younger patients with high CRP peak concentration seemed to have lower mortality. Rapidly decreasing CRP kinetics during the first days of hospital admission predicts hospital survival. However, this benefit gets diminished with the delay until a 50% decrease is achieved, from the second day onwards, with no clear cut-off point. Failure to reduce CRP concentration by at least 50% is associated with higher mortality rates.
